# Canine Influenza Virus A(H3N2) Clade with Antigenic Variation, China, 2016–2017

**DOI:** 10.3201/eid2501.171878

**Published:** 2019-01

**Authors:** Yanli Lyu, Shikai Song, Liwei Zhou, Guoxia Bing, Qian Wang, Haoran Sun, Mingyue Chen, Junyi Hu, Mingyang Wang, Honglei Sun, Juan Pu, Zhaofei Xia, Jinhua Liu, Yipeng Sun

**Affiliations:** China Agricultural University, Beijing, China (Y. Lyu, S. Song, L. Zhou, Q. Wang, Haoran Sun, M. Chen, J. Hu, M. Wang, Honglei Sun, J. Pu, Z. Xia, J. Liu, Y. Sun);; China Animal Disease Control Center, Beijing (G. Bing)

**Keywords:** influenza A virus, dogs, H3N2, antigenicity, viruses, respiratory infections, canine influenza, zoonoses, evolution, adaptation, China, surveillance, PB2, serology, mammalian adaptations, influenza

## Abstract

During 2012–2017, we collected throat swabs from dogs in China to characterize canine influenza virus (CIV) A(H3N2) isolates. A new antigenically and genetically distinct CIV H3N2 clade possessing mutations associated with mammalian adaptation emerged in 2016 and replaced previously circulating strains. This clade probably poses a risk for zoonotic infection.

Canine influenza can be caused by a variety of influenza A viruses, including equine-origin H3N8 and avian-origin H3N2 viruses, which are both established lineages in dogs worldwide. Canine influenza virus (CIV) A(H3N8) has predominantly circulated in the United States since 2004 (*1*,*2*), and CIV A(H3N2) mainly prevails in China and South Korea (*3*,*4*). CIV H3N2 was first isolated in 2006 from Guangdong Province, China, and found to be genetically most closely related to H3N2 avian influenza viruses prevalent in aquatic birds in South Korea (*5*). Since 2006, H3N2 CIV has rapidly become prevalent in China and South Korea (*6*,*7*) and has also been isolated in Thailand and the United States (*8*,*9*).

CIV usually causes mild respiratory symptoms, and CIV-infected dogs often recover without treatment. As a consequence, animal owners and veterinarians often neglect treating CIV infections, creating an opportunity for CIVs to circulate and further adapt in dogs. Mutations leading to better growth in dogs could enhance infectiousness in other mammals (e.g., humans). Also, CIVs are antigenically novel to the human immune system and, thus, might pose a threat to public health. Therefore, we set out to characterize CIV H3N2 in dogs in China to assess the potential risk to the public.

## The Study

During October 2012–July 2017, we collected 399 throat swabs from dogs with respiratory symptoms in pet hospitals and kennels in China to monitor for CIV H3N2 epidemics and virus evolution. We amplified the matrix gene by real-time reverse transcription PCR using Influenza A Virus V8 Rapid Real-Time RT-PCR Detection Kit (Beijing Anheal Laboratories Co., Ltd. http://anheal.company.weiku.com) and isolated and identified virus isolates using methods previously described (*6*). Of 399 samples, 54 (13.5%) contained CIV H3N2 isolates. Of these 54 isolates, 43 were from Beijing, 6 from Nanjing, 3 from Shanghai, and 2 from Xi’an.

**Figure 1:**
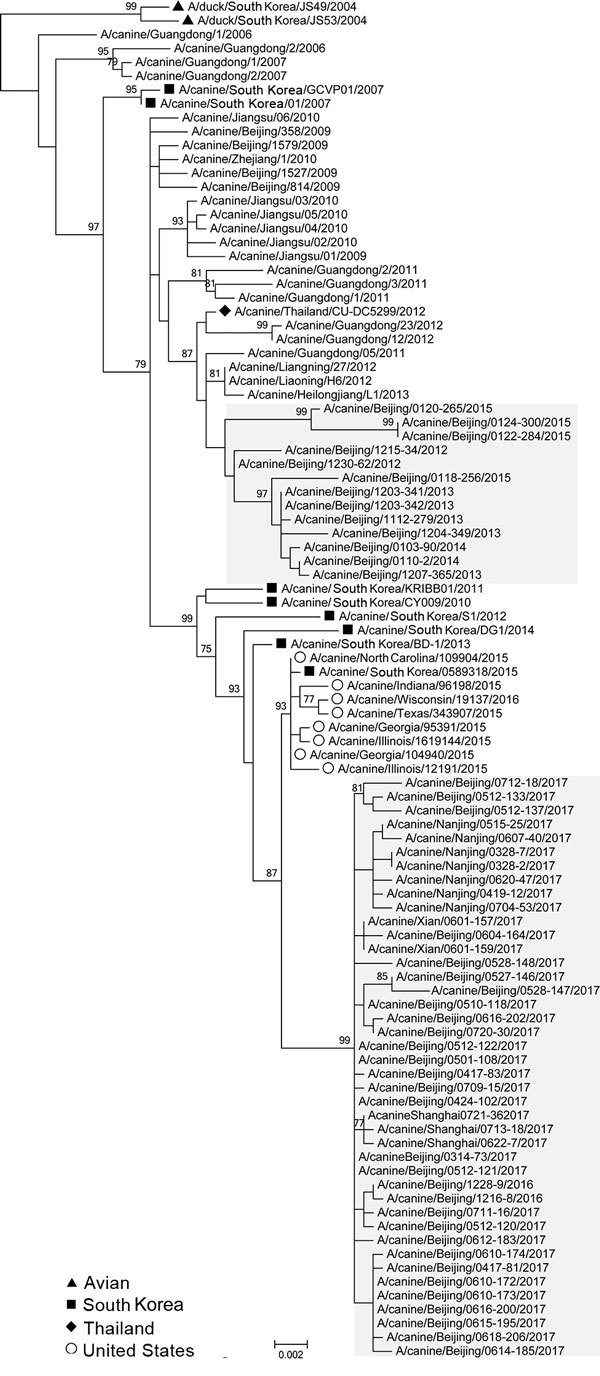
Maximum-likelihood phylogenetic tree of hemagglutinin genomic segment of H3N2 canine influenza viruses (CIVs). The phylogeny of 97 H3N2 CIVs available in public databases and the 8 hemagglutinin genomic segments sequenced in this study were inferred by using MEGA version 6 (https://www.megasoftware.net/) under the general time-reversible plus gamma distribution model with 1,000 bootstrap replicates. Avian isolates of ancestral strain (triangles) and canine isolates from South Korea (squares), Thailand (diamond), and the United States (circles) are indicated. Shading indicates isolates sequenced in this study. Scale bar indicates substitutions per nucleotide.

To characterize the evolution of CIV H3N2, we sequenced the full genome of the 54 isolates (GenBank accession nos. MK212398–829) and performed genetic analyses using available sequences of related viruses from GenBank and the GISAID database (https://www.gisaid.org/). Phylogenetic analysis of worldwide CIV H3N2 isolates indicated that each genome segment of the H3N2 isolates after 2016 formed a separate clade, distinct from other isolates from China, which grouped with isolates from South Korea and the United States (Figure 1; Appendix Figure). Each genome segment of the 41 H3N2 CIVs isolated after 2016 shared high nucleotide sequence identities (99.62% ± 0.09% to 99.88% ± 0.10%). Among these isolates, the time to most recent common ancestor computed by molecular clock analysis (*10*,*11*) was similar for each genome segment; all ancestors dated back to early- to mid-2016 (Appendix Figure). Therefore, the introduction of this CIV H3N2 clade into China most likely occurred in 2016 as either a single event or multiple events involving genetically similar viruses. This clade was more closely related to earlier H3N2 CIVs than the ancestral H3N2 avian influenza viruses from South Korea (Figure 1), and viruses of this clade could have originated from H3N2 CIVs circulating in South Korea or the United States.

We then investigated the molecular characteristics of these viruses. Although all the CIV H3N2 isolates from this clade still possessed 226Q and 228G (which confer specificity to cell entry receptors in birds) in hemagglutinin, they also possessed the 4 amino acid substitutions 251R and 590S in polymerase basic 2 and 146S and 242I in hemagglutinin, which have frequently been identified in human influenza viruses. Of note, 251R and 590S in polymerase basic 2 are known determinants of adaptation to growth in mammals (Table 1) (*12*,*13*).

**Table 1:** Possible mammalian adaptation related to amino acid substitutions in CIV H3N2, China, 2016–2017*

Category	Virus protein, amino acid position, amino acid (frequency, %)				
	Polymerase basic 2			Hemagglutinin	
251	590	146	242
CIV H3N2, 2006–2015	K (89.19), R† (8.11), G (2.70)	G (100)		G (91.89), S (8.11)	V (100)
CIV H3N2, 2016–2017	R† (100)	S† (100)		S (100)	I (100)
Influenza virus A(H3N2) in humans	R† (99.82), K (0.14), G (0.04)	S† (89.76), G (10.06), N (0.14), T (0.04), R (0.01)		S (99.57), G (0.42), F (0.01)	I (99.29), V (0.39), M (0.20), T (0.08), L (0.03), K (0.02)
Influenza A(H1N1)pdm09 virus	R† (98.99), K (0.99), I (0.02)	S† (99.43), N (0.35), G (0.22)		K (99.64), N (0.23), R (0.07), E (0.06)	F (99.99), L (0.01)

*CIV, canine influenza virus. †Known determinant for adaptation to mammals.

**Figure 2:**
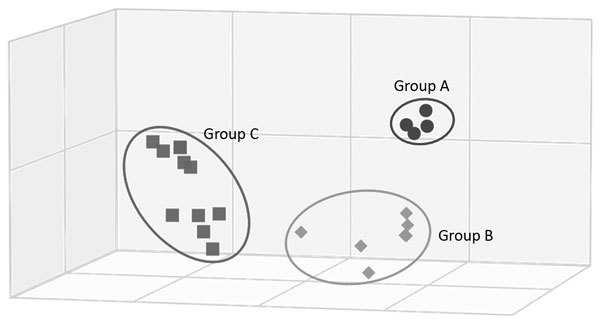
Antigenic cartograph representative of hemagglutinin inhibition (HI) titers of canine influenza viruses, China, 2009–2017, showing antigenic groups A–C. Map was generated by using AntigenMap 3D (http://sysbio.cvm.msstate.edu/software/AntigenMap) and HI data shown in Table 2. One unit (cell) represents a 2-fold change in HI titer.

Antigenic analysis with ferret antiserum against representative viruses of different clades demonstrated a diversity of reaction patterns that generally corresponded with phylogenetic relationships (Table 2). H3N2 CIVs isolated during 2016–2017 reacted well with antiserum against viruses of the same lineage and less well with antiserum against viruses of other lineages. Numeric analysis of these hemagglutinin inhibition (HI) titers with AntigenMap (http://sysbio.cvm.msstate.edu/software/AntigenMap) revealed that H3N2 CIVs isolated after 2016 had a distinguishable antigenic reaction pattern (Figure 2).

**Table 2:** Antigenic analysis of H3N2 subtype canine influenza viruses, China, 2009–2017*

Antigenic group and virus	HI titer, by antigenic group and antiserum							
A, 814/2009	A, 362/2009	B, 0110-2/2014	B, 0118-256/2015	B, 0120-265/2015	C, 1228-9/2016	C, 0512-13720/17	C, 0527-147/2017
A								
814/2009	1,280†	1,280	1,280	160	320	320	320	160
1527/2009	640	1,280	2,560	320	160	320	320	320
1579/2009	640	2,560	1,280	160	160	320	320	320
362/2009	640	1,280†	1,280	320	160	320	160	160
B								
1215-34/2012	160	640	1,280	160	640	1,280	640	1,280
0203-342/2013	320	640	640	640	640	640	1,280	320
1207-365/2013	320	640	1,280	640	1,280	640	1,280	640
0110-2/2014	640	2,560	1,280†	640	320	1,280	640	640
0118-256/2015	320	640	1,280	1,280†	640	640	320	320
0120-265/2015	640	640	1,280	640	1,280†	640	320	640
0124-300/2015	320	1280	1280	640	640	160	640	640
C								
1228-9/2016	80	80	80	160	1,280	1,280†	640	640
0424-102/2017	80	80	320	80	160	640	640	1,280
0512-120/2017	80	80	320	160	320	640	1,280	1,280
0512-133/2017	80	40	320	40	160	640	640	1,280
0512-137/2017	160	80	320	160	320	1,280	1,280†	640
0527-148/2017	80	80	320	80	320	640	2,560	1,280
0527-146/2017	80	40	320	80	320	640	640	640
0527-147/2017	80	320	320	160	160	2,560	1,280	1,280†
0601-159/2017	80	40	320	80	160	640	640	640
0610-173/2017	40	40	320	80	160	640	640	640
0721-36/2017	80	40	320	80	160	640	640	640

*Virus names are abbreviated as serial number and year. HI titers are the inverse of the highest dilution that inhibited hemagglutination. Cells containing moderate HI titers (160, 320) are shaded gray and high titers (>640) black. Low HI titers (40, 80) were not shaded. HI, hemagglutinin inhibition. †Homologous titers.

The CIV H3N2–positive dogs in this study generally had only respiratory symptoms and recovered within 10 days. However, the virus spread rapidly. Among dogs in a cohort, 1 displayed mild disease (cough, runny nose, lethargy) soon after being introduced into a kennel. Within 3 days, similar symptoms were observed in 16 more dogs within that kennel. Real-time reverse transcription PCR confirmed that all 17 dogs were CIV positive. We obtained 2 CIV H3N2 isolates (A/canine/Beijing/0512-133/2017 and A/canine/Beijing/0512-137/2017) from 2 German shepherd dogs in this kennel.

To determine the prevalence of CIV H3N2 in dogs, we randomly performed serologic surveillance for H3N2 virus among dogs visiting the Veterinary Teaching Hospital of China Agricultural University (Beijing, China) in 2017. Of 240 serum samples, 15 (6.3%) were positive for CIV H3N2 (HI titers against A/canine/Beijing/0512-137/2017 of ≥40). To evaluate whether humans can be infected by CIV H3N2, we collected serum samples from pet owners (n = 50), veterinarians (n = 5), and animal hospital staff (n = 23) who had contact with CIV-positive dogs. Serum from 1 pet owner tested positive for CIV H3N2 (HI titer 80), revealing that this virus is a potential threat to public health.

## Conclusions

CIV H3N2 originated from avian influenza viruses in aquatic birds. We found that H3N2 viruses of a novel genetic clade and antigenicity have prevailed in dogs in some areas of China since 2016, completely replacing the previous strains; this H3N2 clade might have originated from CIVs in South Korea or the United States. However, the sparse sequence data for isolates from South Korea and the United States and the absence of CIV H3N2 sequences from these countries after 2016 prevent identification of the ancestor of this clade. Unlike the geographic clustering of isolates observed during the spread of H3N2 CIVs in the United States (*8*), the H3N2 CIVs isolated during 2016–2017 in Beijing (northern China), Shanghai and Nanjing (southeastern China), and Xi’an (western China) have high genetic identities.

In 2017, the percentage of dogs treated at the Veterinary Teaching Hospital of China Agricultural University that were seropositive for CIV H3N2 was 6.3%, higher than the percentage during 2012–2013 (3.5%) (*14*). The wide prevalence and increased seropositivity of H3N2 variants suggest the lineage that emerged in 2016 might possess greater infectivity in dogs than earlier viruses, which might have resulted in clade replacement. The possibility of stochastic events leading to the disappearance of the previous clade should not be excluded. Considering that preadaptation of influenza A(H1N1)pdm09 virus genes to mammalian hosts through prior circulation for several decades in swine might have contributed to the emergence of viruses containing these genes in humans, the potential adaptation of this CIV H3N2 clade to mammals and its public health threat should be further evaluated.

Because dog competitions and trade involving different countries are frequent and the surveillance of CIV is limited, further studies should focus on determining whether viruses of this CIV H3N2 lineage are prevalent in other countries. Global active surveillance to monitor the spread of these viruses among dogs should also be enhanced. Such efforts could prevent further CIV spread and adaptation and will be critical for identifying public health threats that could emerge at the animal–human interface.

Appendix Dated phylogenetic analysis of each genome segment of H3N2 canine influenza viruses, China.
