# Supplementary material for: Canine Influenza Virus A(H3N2) Clade with Antigenic Variation, China, 2016–2017
Source: Emerg Infect Dis. 2019 Jan;25(1):161–5. doi: 10.3201/eid2501.171878 (PMC6302592; doi:10.3201/eid2501.171878)
Supplement: Appendix — Dated phylogenetic analysis of each genome segment of H3N2 canine influenza viruses, China. [file 17-1878-Techapp-s1.pdf]

Article DOI: https://doi.org/10.3201/eid2501.171878

Canine Influenza Virus A(H3N2) Clade with Antigenic Variation, China, 2016–2017

Appendix

Appendix Figure (following pages). Dated phylogenies of the genome segments of H3N2 canine influenza viruses, China. The inferred most recent common ancestors of H3N2 canine influenza viruses isolated during 2016–2017 are indicated by blue dots. Timeline is in years. A) Polymerase basic 2; B) polymerase basic 1; C) polymerase acidic; D) hemagglutinin; E) nucleoprotein; F) neuraminidase; G) matrix; H) nonstructural protein.

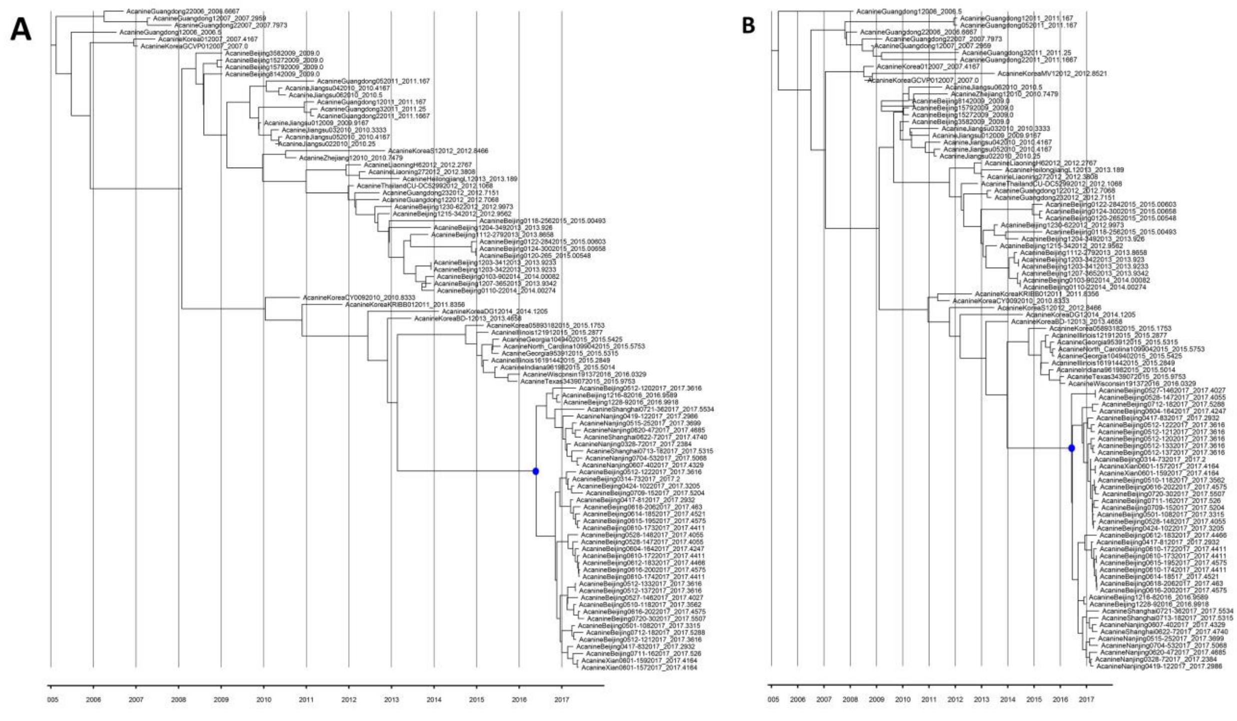

C

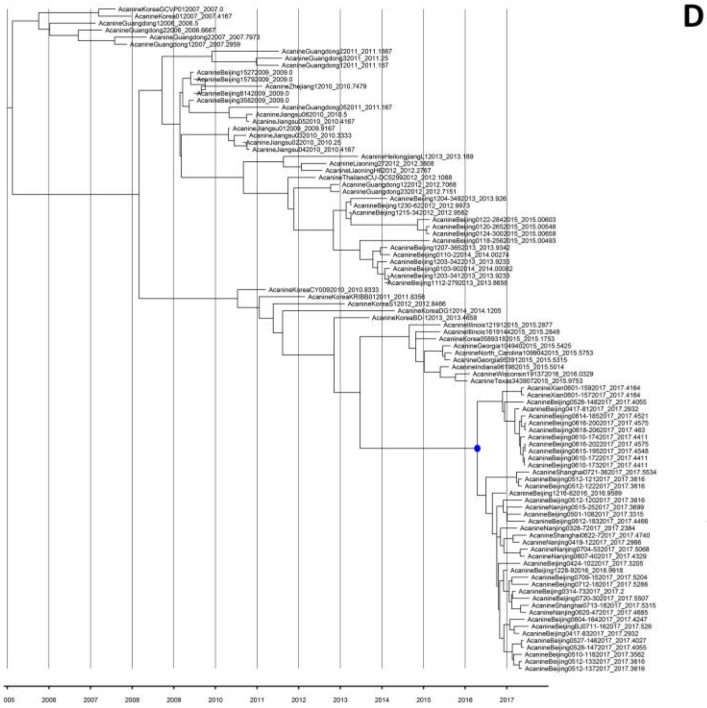

D

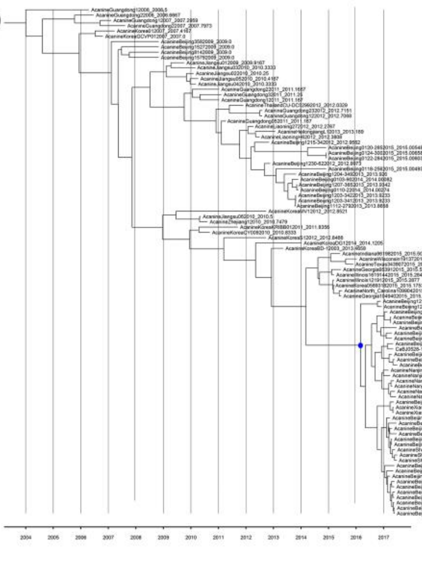

E

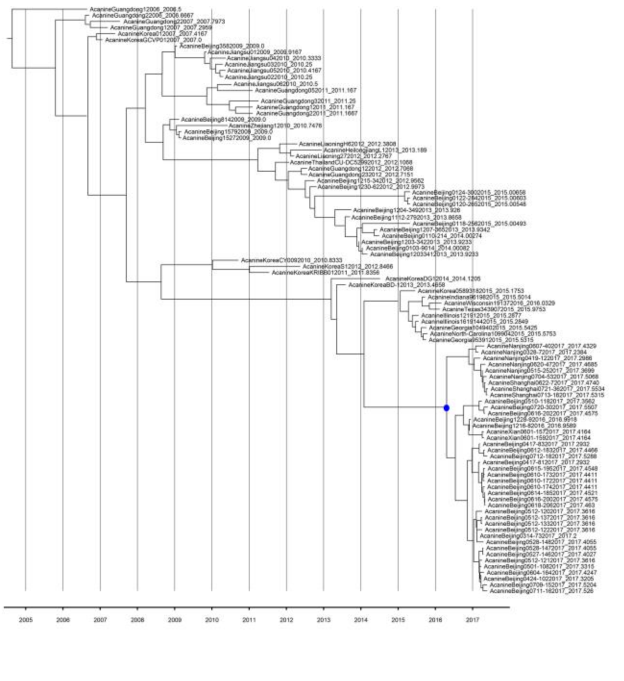

F

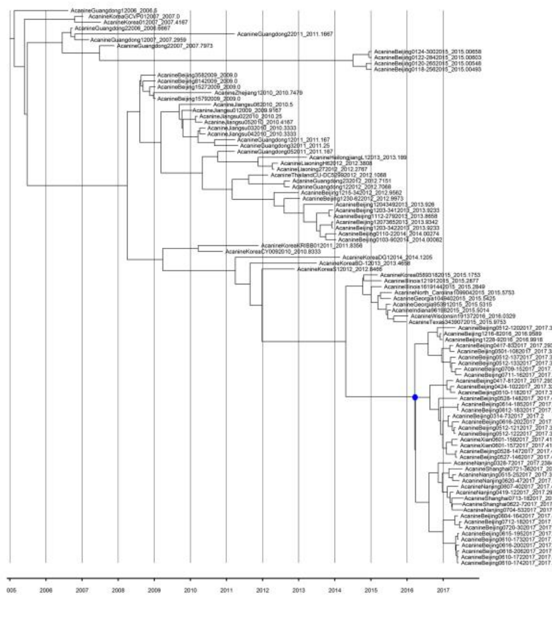

G

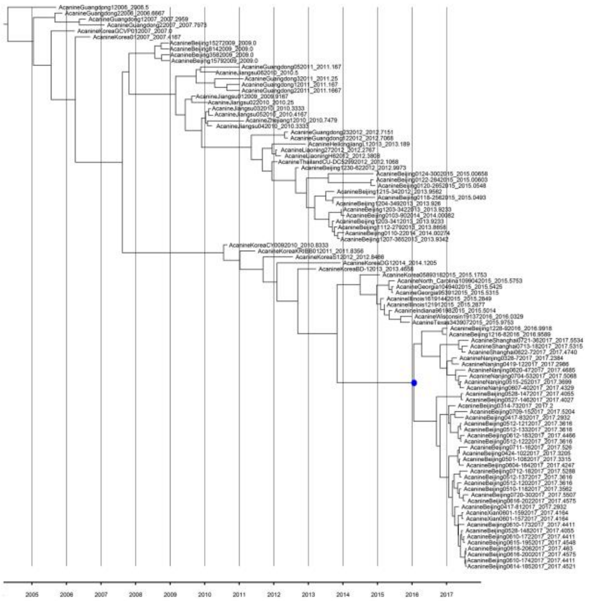
2005
2006
2007
2008
2009
2010
2011
2012
2013
2014
2015
2016
2017

H

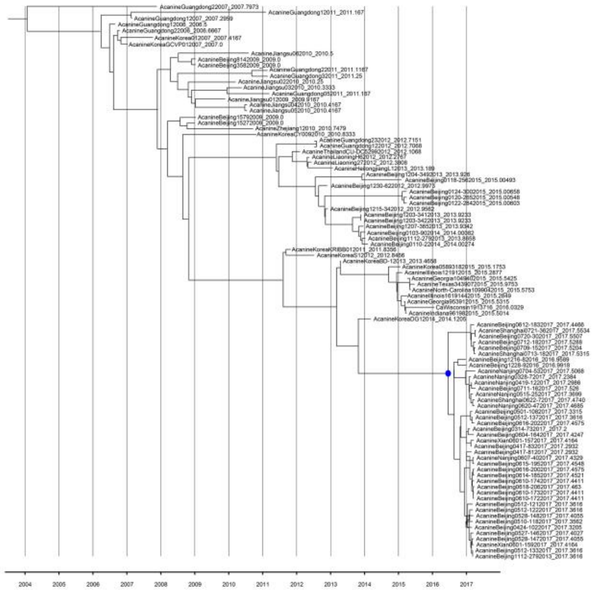
2004
2005
2006
2007
2008
2009
2010
2011
2012
2013
2014
2015
2016
2017
